# Nutrient Inadequacy Rates Among Japanese Adolescents Aged 10–14: Cross-Sectional Pooled Analysis From 2018 to 2023 (NICE EVIDENCE Study 5)

**DOI:** 10.1155/jnme/5568303

**Published:** 2025-07-24

**Authors:** Efrem d'Avila Ferreira, Sakiko Yoshizawa Morikawa, Yasunaga Takeda, Izumi Ikeda, Risa Igarashi Matsumoto, Mizuki Takeuchi, Mariko Hatta, Chika Horikawa, Laymon Khin, Hajime Ishiguro, Kazuya Fujihara, Yohei Ogawa, Hirohito Sone

**Affiliations:** ^1^Department of Hematology, Endocrinology and Metabolism, Faculty of Medicine, Niigata University, Niigata, Japan; ^2^Department of Food and Nutrition, Faculty of Human Life Sciences, Tokushima Bunri University, Tokushima, Japan; ^3^Department of Health and Nutrition, Niigata University of Health and Welfare, Niigata, Japan; ^4^Department of Health and Nutrition, Faculty of Human Life Studies, University of Niigata Prefecture, Niigata, Japan; ^5^Department of Pediatrics, Faculty of Medicine, Niigata University, Niigata, Japan

**Keywords:** adolescents, dietary adherence, nutritional adequacy

## Abstract

This study aimed to describe the demographics, nutrient and food group intakes, and adherence to dietary guidelines among Japanese adolescents aged 10–14 years. This cross-sectional study involved 5225 elementary and junior high school students who were surveyed for the NICE EVIDENCE project from 2018 to 2023 (mean age: 11.9 ± 1.4 years; 50.3% male). Nutritional intake was assessed using the brief-type self-administered diet history questionnaire (BDHQ). Nutrient intake inadequacy was assessed in four groups (males and females in the 10-11- and 12–14-year age groups) based on the Dietary Reference Intakes for Japanese (2025). Demographic data indicated that the prevalence of thinness was higher among females, while the prevalence of overweight and obesity was higher among males. Overall, daily breakfast consumption was high but tended to decrease with age. Nutritional inadequacy rates were generally higher in females, particularly in the 12–14-year age group. Major micronutrient inadequacies included iron, calcium, magnesium, manganese, phosphorus, potassium, vitamin D, and vitamin B6. Among the nutrients with established dietary goals, salt equivalent intake exceeded 90% inadequacy in all groups, followed by intake inadequacies in dietary fiber, fat energy percentage, and potassium. Confectionery consumption was relatively high in this population (> 50 g/day), indicating a potential area for dietary improvement. Promoting regular breakfast and increasing micronutrients and fiber intakes, while reducing sodium, total fat, and saturated fat intakes, could address nutritional inadequacies in this population.

## 1. Introduction

Adolescence is the developmental period between childhood and adulthood, typically ranging from ages 10–19 years. During this time, individuals experience significant physical, psychological, and social changes, which impact dietary habits and nutritional needs [1]. Adolescence includes early, middle, and late stages. Ages 10–14, or early adolescence, involve major changes and are considered an overlooked period in research [2]. It also represents a critical window for changes in cognition, motivation, and social behavior, with long-term implications for health and lifestyle habits [3]. Following dietary guidelines based on the regional diet of a respective area is essential for ensuring that adolescents meet their dietary requirements during this critical stage of development. Traditional regional diets are viewed as sustainable dietary patterns and have been shown to improve cardiovascular risk factors [4].

In Japan, long-term analyses of National Health and Nutrition Survey data have shown declining intakes of key vitamins and minerals among children and adolescents, alongside persistently high sodium intake [5]. Fruit and vegetable consumption remains below recommendations, due in part to a lack of habit of eating fruit in the morning and low awareness of vegetable intake recommendations [6]. Similar trends of poor adherence to dietary guidelines are seen in countries such as China and the United States [7, 8]. Japanese school lunches, regulated by government standards, are designed to provide at least one-third of children's daily energy needs and meet specific minimum requirements for key nutrients such as vitamins A (≥ 40%), B_1_ (≥ 40%), and B_2_ (≥ 40%); calcium (≥ 50%); iron (≥ 40%); and fiber (≥ 40%) [9]. School lunches in Japan have improved nutrient intakes but do not fully address issues such as excessive fat and salt consumption and insufficient fiber intake [10].

Early adolescents (ages 10–14) are particularly vulnerable to nutrient deficiencies compared to older adolescents, with higher levels of micro- and macronutrient intake inadequacies [11]. However, studies on this specific age group are limited, leaving gaps in our understanding of their unique dietary behaviors and nutritional statuses.

To address these gaps, this study focuses on describing the demographics, nutrient and food group intakes, and adherence to dietary guidelines of Japanese adolescents aged 10–14 years. By identifying nutrient intake gaps in this group, the study aims to inform early interventions that can prevent future health issues.

## 2. Methods

### 2.1. Study Population and Design

This cross-sectional study involved elementary and junior high school students from the cities of Agano and Sanjo in Niigata Prefecture, who were surveyed annually for the Niigata Screening for Preventing the Development of Noncommunicable Disease (NICE EVIDENCE project) from 2018 to 2023. The project aims to prevent noncommunicable diseases. The initial cohort included 8098 participants. First-grade junior high school students surveyed in 2020 and 2021 (*N* = 1576) were excluded because they had already participated in the survey two years earlier as fifth-grade elementary school students. Further exclusions were made for participants below the age of 10 (*N* = 1), those with energy intakes below 600 kcal/day or above 4000 kcal/day (*N* = 165), and those with missing data for physical activity (*N* = 1104) and breakfast consumption (*N* = 27), resulting in a final sample of 5225 participants. The selected energy intake cutoffs were applied to exclude possible under- or overreporting, following a previous study that utilized the same database [12]. On weekdays, all adolescents were provided with school lunches designed based on dietary guidelines.

### 2.2. Anthropometric, Dietary, and Lifestyle Assessments

Body height (cm) was recorded to the nearest 0.1 cm with participants standing barefoot. Body weight (kg) was measured to the nearest 0.1 kg, with participants wearing light indoor clothing. Adolescents' weight status was assessed using the International Obesity Task Force age- and sex-specific cutoff, which is linked to the adult BMI cutoff values for thinness (< 18.5 kg/m^2^), normal weight (18.5–24.9 kg/m^2^), overweight (≥ 25 kg/m^2^), and obesity (≥ 30 kg/m^2^) [13]. Diet, physical activity, and demographic variables were self-reported, based on a questionnaire completed by the children or their guardians. Responses were checked for missing values and obvious errors, and resurveys were conducted as part of the planned protocol when such issues were identified. Physical activity was measured in metabolic equivalents (METs) using the Japanese International Physical Activity Questionnaire (IPAQ) for early adolescents [14]. Nutritional intake was assessed using the brief-type self-administered diet history questionnaire (BDHQ) [15]. Both tools are validated for Japanese children. The BDHQ is a fixed-portion food frequency questionnaire, which reduces respondent burden by not requiring portion size reporting. Food intake is later estimated using predefined sex- and age-specific standard portion sizes to improve accuracy. The calculation is updated as needed in response to new scientific evidence regarding dietary behavior and intake estimation methods. Nutrient intake was calculated from the Standard Tables of Food Composition in Japan 2015 (Seventh Revised Edition) [16]. Nutrient adequacy was based on the Report of the Study Group on the Formulation of the Dietary Reference Intakes for Japanese (2025 Edition), by the Ministry of Health, Labor and Welfare [17]. Commuting methods were assessed as the primary modes of transportation used over the past month and included the options of walking, cycling, or using a bus/car. Breakfast consumption was assessed with the following options: “Eat daily,” “There are days I do not eat,” “There are many days I do not eat,” and “Do not eat.”

### 2.3. Statistical Analysis

Participants were stratified into four groups based on the 2025 Dietary Reference Intakes for Japanese, by the Ministry of Health, Labor and Welfare, which separates guidelines by age and gender: males aged 10-11 years, females aged 10-11 years, males aged 12–14 years, and females aged 12–14 years. Categorical variables were presented as numbers and percentages and compared using the chi-square test. Nutrient inadequacy rates were calculated by comparing intakes against age- and sex-specific estimated average requirement (EAR), adequate intake (AI), and dietary goal (DG) values, listed in Supporting Table 1. Shapiro–Wilk tests showed energy and macronutrient data were non-normal; thus, nonparametric tests were used. Continuous variables were reported as means with standard deviations, except for physical activity, which was presented as the median with interquartile range due to its skewness. Continuous data were analyzed using the Mann–Whitney *U* test to compare two groups, and the Kruskal–Wallis test was used to assess differences among three or more groups. Values of *p* < 0.05 were considered statistically significant. All tests were performed using IBM SPSS Statistics Version 27.

## 3. Results

### 3.1. Characteristics of Study Participants

Basic characteristics of study participants, both overall and stratified by age and gender, are described in Table 1. The prevalence of overweight and obesity was higher in males, while the prevalence of thinness was higher in females. Physical activity was highest among males aged 12–14 years and lowest among females aged 10-11 years. All groups achieved a moderate level of physical activity, with total activity reaching at least 600 MET-minutes per week. Daily breakfast consumption frequency tended to be higher in younger adolescents compared to older ones. Walking was the predominant commuting method across all groups, with the proportion of bicycle commuting tending to increase with age.

### 3.2. Food Group Intakes

Food group consumption by sex and age, along with *p* values for sex differences within the 10-11- and 12–14-year age groups, are presented in Table 2.

#### 3.2.1. Age Group 10-11 Years

Males consumed significantly more cereals, potatoes, sugar/sweeteners, soybeans, other vegetables, seafood, meat, dairy, fats and oils, nonalcoholic beverages, and condiments/spices than females. Females consumed more eggs. There were no significant intake differences in green and yellow vegetables, fruits, and confectionery between groups.

#### 3.2.2. Age Group 12–14 Years

Males had higher intakes of all food groups, except for green and yellow vegetables and fruits, for which no significant differences were observed.

### 3.3. Inadequacy Levels of Energy, Protein, and Nutrients With DGs

Table 3 shows the mean nutrient intakes and inadequacy levels by sex and age group. More than half of the individuals in each group had an energy intake below the estimated requirements for moderate physical activity. Protein intake met the EAR in the majority of participants across all groups. Among the nutrients with tentative DGs for preventing lifestyle-related diseases, salt equivalent intake showed the highest level of intake inadequacy. Salt equivalent intake exceeded the dietary guidelines in over 90% of study subjects across all groups. Energy from fat and saturated fat had the highest level of intake inadequacy for macronutrients, with intakes above the DG recommendations. Dietary fiber also showed high intake inadequacy levels, which increased with age, and failed to meet the DG in all four groups.

### 3.4. Inadequacy Levels of Micronutrients

Micronutrient inadequacy levels were higher in the older groups. Only copper showed less than 10% levels of intake inadequacy in all groups.

#### 3.4.1. Age Group 10-11 Years

For males, the most inadequate micronutrient intakes were vitamin D (40.5%), phosphorus (40.4%), and manganese (37.7%). In females, the most inadequate micronutrient intakes were iron (74.8%, based on requirements for menstruating individuals), vitamin D (51.9%), and calcium (48.4%).

#### 3.4.2. Age Group 12–14 Years

For males, the most inadequate micronutrient intakes were calcium (57.6%), vitamin B6 (47.3%), and magnesium (43.5%). In females, the micronutrients with the highest levels of inadequate intake were iron (78.7%, based on requirements for menstruating individuals), calcium (61.2%), and magnesium (59.9%).

### 3.5. Energy, Macronutrient Proportions, and Dietary Fiber According to Body Weight Status

Table 4 shows the differences in nutrient intakes according to body weight status. Energy intake was significantly higher in individuals with normal weight compared to those with thinness, overweight, and obesity. Individuals with overweight and obesity had significantly higher energy intake from protein. There were no significant differences in the proportion of energy from fat or carbohydrate among the groups. Dietary fiber intake was highest in normal weight individuals, with the lowest intake observed in those with overweight.

### 3.6. Energy, Macronutrient Proportions, and Dietary Fiber According to Daily Breakfast Consumption Status

Table 5 presents the differences in nutrient intakes based on daily breakfast consumption status. Energy intake, total fiber, and energy intake from protein were significantly higher in daily breakfast consumers, while the proportion of energy from carbohydrates was higher in nondaily breakfast consumers. Energy intake from fat did not differ significantly between the groups.

## 4. Discussion

Our analysis of nutritional intake among 5225 Japanese early adolescents reveals age- and gender-specific inadequacies not reported in previous studies in Japan. Specifically, the 12–14-year age group showed a higher prevalence of intake inadequacy compared to the 10-11-year age group. Notably, there were considerable intake inadequacies in dietary fiber, salt equivalent, and percentage of energy from fat and saturated fat, as well as in various micronutrients, including iron, calcium, magnesium, phosphorus, potassium, vitamin D, and vitamin B6. Moreover, adolescent females tended to have higher levels of nutritional inadequacy compared to adolescent males, particularly for iron, calcium, magnesium, and vitamin D.

These findings are in line with studies from Japan [18] and other countries [19–21], which also reported inadequate intakes of calcium, iron, potassium, magnesium, vitamins, sodium, and dietary fiber among adolescents. In Japanese children and adolescents, the intake of fish and shellfish, traditionally important sources of essential nutrients, is declining due to lifestyle changes and convenience-driven food choices. Meanwhile, meat consumption increased, while fruit and milk intake decreased between 2001 and 2019 [22]. Ultra-processed foods, which are often high in unhealthy fats, sodium, and added sugars, likely contributed to these imbalanced dietary patterns [23].

One possible explanation for the higher nutrient inadequacy levels in the 12–14-year age group compared to the 10-11-year age group could be their lower, yet still over 90%, daily breakfast consumption rate. In the current study, adolescents who reported daily breakfast consumption had higher energy, total fiber, and relative protein intakes compared to nondaily breakfast consumers, consistent with findings from Canadian children and adolescents [24]. In addition, because underreporting is more common among individuals with overweight or obesity, the use of self-reported data may have led to an overestimation of nutrient inadequacy, especially since obesity rates were higher in the 12–14-year age group [25].

Intakes of iron, calcium, magnesium, and vitamin D were particularly inadequate in girls. Incorporating iron-rich foods (e.g., lean meats and legumes), calcium (e.g., green and yellow vegetables, fish, soybeans, and dairy products), magnesium (e.g., grains, legumes, vegetables, seafood, seaweed, and fruits), and vitamin D sources (e.g., fortified dairy products and fish) into their diet can help address these deficiencies and improve overall nutritional status.

Although menstrual status was not assessed, iron intake inadequacy was notably higher when evaluated against dietary reference values for menstruating females, which assume greater physiological requirements. A decreasing trend in overall iron intake has been shown among children and adolescents in Japan [5]. Fortified cereals, an important iron source in other countries, are not common in Japan [18]. These factors may contribute to an increased risk of iron inadequacy.

Calcium intake in the Japanese diet mainly comes from plant and fish-based sources, which provide a lower-fat alternative to dairy and may help address the high-fat inadequacy observed in this population [26]. In our population, calcium inadequacy rates were lowest in 10-11-year-old boys (36.2%) and highest in 12–14-year-old girls (61.2%). Calcium requirements peak in the 12–14-year-old group according to the Japanese dietary reference values, highlighting the importance of adequate calcium intake during this period.

The prevalence of vitamin D inadequacy was lowest in 10-11-year-old boys (40.5%) and highest in 12–14-year-old girls (56.4%). In Japan, a middle-latitude country, blood vitamin D levels vary seasonally, with deficiency rates of 47.7% in summer and 82.2% in winter [27]. Fish is the main dietary source, followed by eggs. Moderate sun exposure was shown to effectively compensate for vitamin D deficiencies in individuals with inadequate dietary intake of this nutrient [28].

Sodium, measured by salt equivalents, showed the highest level of intake inadequacy among adolescents, with levels exceeding the recommended guidelines. While the traditional Japanese diet was associated with a high nutrient density, it was also linked to elevated sodium intake [29]. However, the traditional Japanese diet was reported to be inversely correlated with the sodium-to-potassium ratio [29, 30]. A systematic review indicated that the ratio was more strongly associated with blood pressure outcomes than either sodium or potassium alone in adults with hypertension [31]. The review noted a gap in research on the relationship between the sodium-to-potassium ratio and blood pressure in children. Given the rise in childhood obesity in Japan over the past decade [32], exploring the potential impact of this ratio warrants further study.

In our study population, there was a high inadequacy of fiber intake. Dietary fiber is defined as nondigestible carbohydrates, including lignin, that pass to the large intestine, and it is essential for maintaining bowel health [33]. Higher intake of fiber from fruits, beans, and vegetables, but not from grains, showed an inverse relationship with overall mortality in Japanese adults [34]. Increasing fiber intake from fruits, beans, and vegetables, along with incorporating other sources such as nuts, seeds, seaweed, and whole grains, not only enhances fiber adequacy but also improves overall nutrient intake.

Compared to national estimates from 2019 [22], adolescents from our study appeared to have higher intake of seafood, fruit, and confectionery and substantially lower consumption of meat. These differences likely reflect regional dietary habits, food environments, and school meal programs. Such regional dietary patterns should be considered when interpreting our findings. In particular, the higher confectionery intake may contribute to a dietary pattern characterized by increased consumption of saturated fat and sugars, which is energy-dense yet poor in micronutrients [35].

The Japanese school lunch program, designed to meet key nutrient requirements, plays a significant role in mitigating nutrient inadequacies. While school lunches are important, additional strategies are needed to enhance dietary adequacy. In this regard, nutrition education programs incorporating either theoretical classes [36] or practical activities such as gardening and cooking [37] have been shown to effectively improve children's diet.

The strengths of this study include a large sample size, comprehensive characterization of adequacy levels, and a focus on the early adolescent population, who are at a critical period for lifelong habit formation. Study limitations include the reliance on self-reported data for nutritional intake, the use of a BDHQ (a short, fixed-portion questionnaire with reasonable ranking ability but limited accuracy for absolute nutrient intake), potential seasonal and day-to-day variations in dietary intake, and the limited regional scope as the study is restricted to two cities in Niigata Prefecture, Japan. Nonetheless, these two mid-sized cities are potentially representative of many similar communities in Japan. In addition, 1131 participants were excluded due to missing data. Energy intakes did not differ significantly between the included (2112.8 ± 601.2 kcal/day) and excluded (2130.4 ± 638.6 kcal/day) participants (*p*=0.569). However, significant differences in age and BMI (both *p* < 0.001) were observed, suggesting potential systematic differences between excluded and included participants. While some bias due to exclusion is possible, the large sample size likely minimizes its impact on the findings.

## 5. Conclusions

We found a high frequency of micronutrient intake inadequacies among Japanese adolescents aged 10–14 years, with higher prevalence in the 12–14-year age group and in females. Promoting regular breakfast consumption and increasing overall intake of micronutrients, particularly iron, calcium, vitamin D, magnesium, manganese, phosphorus, and vitamin B6, while also increasing fiber and reducing sodium, total fat, and saturated fat intake, could help address these nutritional intake inadequacies in this population. High confectionery consumption in our population can add sugars and saturated fat and provide little nutritional value, indicating a potential area for dietary improvement. Further research is needed to determine the clinical impact of these intake deficiencies in adolescents.

## Figures and Tables

**Table 1:** Demographic characteristics of study participants overall and by sex and age group.

Variables	All participants (*N* = 5225)	Male 10-11 years (*N* = 1367)	Female 10-11 years (*N* = 1346)	*p*	Male 12–14 years (*N* = 1263)	Female 12–14 years (*N* = 1249)	*p*
Age (years)	11.9 (1.4)	10.6 (0.3)	10.6 (0.3)	0.554	13.3 (0.6)	13.3 (0.6)	0.673
Body weight status (*n*, %)				**< 0.001**			**< 0.001**
Thinness	632 (12.1)	132 (9.7)	223 (16.6)		125 (9.9)	152 (12.2)	
Normal weight	3932 (75.3)	1022 (74.8)	984 (73.1)		947 (75.0)	979 (78.4)	
Overweight	548 (10.5)	171 (12.5)	118 (8.8)		154 (12.2)	105 (8.4)	
Obesity	113 (2.2)	42 (3.1)	21 (1.6)		37 (2.9)	13 (1.0)	
Physical activity (MET-minutes/week)	1710.0 (613.5–4800.0)	1920.0 (780.0–4278.0)	1155.5 (592.8–2346.0)	**< 0.001**	3516.0 (699.0–6495.0)	1612.5 (495.0–5859.0)	**< 0.001**
Consuming breakfast every day (*n*, %)	4891 (93.6)	1318 (96.4)	1297 (96.4)	0.510	1143 (90.5)	1133 (90.7)	0.454
Residential area (*n*, %)				—			0.086
Sanjo	3692 (70.7)	1367 (100.0)	1346 (100.0)		475 (37.6)	504 (40.4)	
Agano	1533 (29.3)	0	0		788 (62.4)	745 (59.6)	
School grade (*n*, %)				—			0.061
5^th^ grade elementary school	2713 (51.9)	1367 (100.0)	1346 (100.0)		0	0	
1^st^ grade junior high school	885 (16.9)	0	0		426 (33.7)	459 (36.7)	
2^nd^ grade junior high school	1627 (31.1)	0	0		837 (66.3)	790 (63.3)	
Year (*n*, %)				0.051			**0.003**
2018	1234 (23.6)	273 (20.0)	298 (22.1)		301 (23.8)	362 (29.0)	
2019	1287 (24.6)	305 (22.3)	311 (23.1)		323 (25.6)	348 (27.9)	
2020	1059 (20.3)	368 (26.9)	386 (28.7)		157 (12.4)	148 (11.8)	
2021	1083 (20.7)	421 (30.8)	351 (26.1)		176 (13.9)	135 (10.8)	
2022	291 (5.6)	0 (0)	0 (0)		166 (13.1)	125 (10.0)	
2023	271 (5.2)	0 (0)	0 (0)		140 (11.1)	131 (10.5)	

*Note:* Data are presented as mean (±standard deviation) for age and median (interquartile range) for physical activity and *n* (%). Significant differences are highlighted in bold (*p* < 0.05).

**Table 2:** Food group intake by sex and age group.

Food groups	Male 10-11 years (valid *N* = 1342)	Female 10-11 years (valid *N* = 1323)	*p*	Male 12–14 years (valid *N* = 1252)	Female 12–14 years (valid *N* = 1249)	*p*
Cereals (g/day)	456.6 (208.7)	376.3 (163.9)	**< 0.001**	538.8 (232.0)	407.5 (186.5)	**< 0.001**
Potatoes (g/day)	38.8 (27.2)	34.5 (25.7)	**< 0.001**	40.2 (31.3)	34.8 (26.6)	**< 0.001**
Sugar/sweeteners (g/day)	3.0 (1.7)	2.8 (1.6)	**0.001**	2.8 (1.7)	2.5 (1.6)	**< 0.001**
Soybeans (g/day)	54.3 (38.6)	48.3 (35.6)	**< 0.001**	53.9 (38.6)	46.7 (34.6)	**< 0.001**
Green and yellow vegetables (g/day)	88.2 (63.4)	83.7 (56.4)	0.155	93.6 (66.0)	89.3 (61.6)	0.116
Other vegetables (g/day)	135.3 (90.4)	122.5 (79.4)	**< 0.001**	136.4 (88.8)	124.9 (76.4)	**0.008**
Fruits (g/day)	89.6 (94.3)	85.0 (88.9)	0.133	92.3 (108.1)	87.7 (103.2)	0.260
Seafood (g/day)	60.6 (40.2)	54.0 (35.3)	**< 0.001**	61.5 (44.6)	52.8 (36.4)	**< 0.001**
Meat (g/day)	82.4 (43.3)	71.4 (37.3)	**< 0.001**	88.6 (44.3)	74.4 (39.6)	**< 0.001**
Eggs (g/day)	33.1 (24.0)	34.1 (20.9)	**< 0.001**	36.7 (26.3)	35.2 (20.9)	**< 0.001**
Dairy (g/day)	312.7 (212.8)	250.5 (177.8)	**< 0.001**	379.5 (265.1)	268.7 (204.0)	**< 0.001**
Fats and oils (g/day)	14.6 (6.3)	12.9 (5.7)	**< 0.001**	15.3 (6.8)	13.3 (6.1)	**< 0.001**
Confectionery^a^ (g/day)	59.0 (40.5)	57.0 (39.3)	0.118	55.6 (43.2)	51.5 (40.7)	**0.011**
Nonalcoholic beverages (g/day)	435.6 (298.4)	412.6 (263.3)	**0.013**	529.6 (367.6)	457.0 (309.8)	**< 0.001**
Condiments and spices (g/day)	321.1 (174.8)	280.5 (149.7)	**< 0.001**	346.6 (177.1)	315.5 (160.2)	**< 0.001**

*Note:* Data are presented as mean (±standard deviation). Valid N: number of participants with available data for all food groups. Significant differences are highlighted in bold (*p* < 0.05).

^a^Confectionery includes Japanese and Western sweets, rice crackers, and ice cream.

**Table 3:** Energy, nutrient intakes, and inadequacy levels by sex and age group.

Nutrient	Male 10-11 years intake (*N* = 1367) Mean (SD)	Female 10-11 years intake (*N* = 1346) Mean (SD)	Male 12–14 years intake (*N* = 1263) Mean (SD)	Female 12–14 years intake (*N* = 1249) Mean (SD)	Male 10-11 years inadequacy (*N* = 1368) *n* (%)	Female 10-11 years inadequacy (*N* = 1346) *n* (%)	Male 12–14 years inadequacy (*N* = 1263) *n* (%)	Female 12–14 years inadequacy (*N* = 1249) *n* (%)
Energy^a^ (kcal/day)	2208.2 (569.7)	1918.7 (496.8)	2364.5 (643.9)	1963.1 (579.1)	782 (57.2)	899 (66.8)	833 (66.0)	992 (79.4)
Nutrients without DRI								
Fat (g/day)	72.9 (24.2)	65.0 (21.8)	77.2 (27.0)	64.8 (23.0)	—	—	—	—
SFA (g/day)	24.5 (9.2)	21.9 (8.1)	26.2 (10.1)	21.9 (8.6)	—	—	—	—
Carbohydrate (g/day)	289.9 (101.3)	243.9 (88.0)	326.5 (113.0)	256.7 (92.3)	—	—	—	—
Sodium (mg/day)	4587.0 (1533.1)	4069.3 (1411.7)	4865.2 (1471.9)	4203.4 (1322.1)	—	—	—	—
Nutrients with EAR								
Protein (g/day)	75.6 (24.6)	66.6 (21.6)	82.0 (27.3)	67.1 (22.9)	77 (5.6)	88 (6.5)	110 (8.7)	184 (14.7)
Vitamin A^b^ (μg/day)	641.1 (362.9)	581.9 (374.4)	691.3 (384.8)	595.7 (326.3)	424 (31.0)	409 (30.4)	523 (41.4)	556 (44.5)
Vitamin B_1_ (mg/day)	0.8 (0.2)	0.7 (0.2)	0.9 (0.3)	0.7 (0.2)	355 (26.0)	343 (25.5)	431 (34.1)	527 (42.2)
Vitamin B_2_ (mg/day)	1.5 (0.5)	1.4 (0.4)	1.7 (0.6)	1.4 (0.5)	220 (16.1)	338 (25.1)	334 (26.4)	459 (36.7)
Niacin (mg/day)	14.8 (5.5)	13.1 (5.0)	15.8 (6.3)	13.4 (5.3)	279 (20.4)	327 (24.3)	319 (25.3)	550 (44.0)
Vitamin B_6_ (mg/day)	1.2 (0.4)	1.1 (0.3)	1.2 (0.4)	1.1 (0.4)	266 (19.5)	618 (45.9)	597 (47.3)	692 (55.4)
Vitamin B_12_ (μg/day)	7.6 (4.3)	6.7 (3.7)	8.0 (4.6)	6.5 (3.8)	93 (6.8)	136 (10.1)	174 (13.8)	312 (25.0)
Folate (μg/day)	319.3 (137.7)	292.3 (129.0)	332.7 (142.7)	298.1 (123.5)	95 (6.9)	123 (9.1)	147 (11.6)	208 (16.7)
Vitamin C (mg/day)	103.2 (55.2)	99.1 (49.2)	108.0 (55.6)	100.5 (51.5)	268 (19.6)	255 (18.9)	337 (26.7)	415 (33.2)
Calcium (mg/day)	737.7 (342.7)	638.3 (294.0)	839.4 (397.3)	662.1 (316.1)	495 (36.2)	652 (48.4)	728 (57.6)	764 (61.2)
Magnesium (mg/day)	251.6 (92.6)	223.6 (83.1)	274.7 (100.7)	229.7 (85.9)	237 (17.3)	385 (28.6)	549 (43.5)	748 (59.9)
Iron (mg/day)	7.9 (2.6)	7.2 (2.3)	8.2 (2.9)	7.2 (2.5)	387 (28.3)	Nonmenstrual criteria: 534 (39.7) Menstrual criteria: 1007 (74.8)	535 (42.4)	Nonmenstrual criteria: 536 (42.9) Menstrual criteria: 983 (78.7)
Zinc (mg/day)	9.4 (3.0)	8.2 (2.5)	10.3 (3.2)	8.3 (2.6)	94 (6.9)	144 (10.7)	161 (12.7)	296 (23.7)
Copper (mg/day)	1.2 (0.3)	1.1 (0.3)	1.3 (0.4)	1.1 (0.3)	8 (0.6)	16 (1.2)	51 (4.0)	48 (3.8)
Nutrients with AI								
*n* − 6 PUFA (g/day)	12.9 (4.1)	11.3 (3.8)	13.3 (4.7)	11.2 (4.0)	200 (14.6)	356 (26.4)	409 (32.4)	637 (51.0)
*n* − 3 PUFA (g/day)	2.7 (0.9)	2.3 (0.8)	2.7 (1.1)	2.3 (0.8)	148 (10.8)	266 (19.8)	383 (30.3)	302 (24.2)
Vitamin D (μg/day)	10.6 (7.0)	9.2 (6.3)	12.0 (8.6)	9.7 (6.6)	553 (40.5)	699 (51.9)	544 (43.1)	704 (56.4)
Vitamin E (mg/day)	8.2 (2.7)	7.5 (2.5)	8.6 (3.2)	7.5 (2.7)	127 (9.3)	271 (20.1)	320 (25.3)	382 (30.6)
Vitamin K (μg/day)	269.7 (156.4)	239.3 (140.7)	270.0 (163.1)	236.5 (137.8)	143 (10.5)	275 (20.4)	254 (20.1)	360 (28.8)
Pantothenic acid (mg/day)	7.3 (2.2)	6.3 (1.9)	7.9 (2.7)	6.5 (2.1)	380 (27.8)	615 (45.7)	484 (38.3)	565 (45.2)
Potassium (mg/day)	2441.5 (984.1)	2189.1 (877.6)	2662.9 (1065.0)	2241.8 (905.3)	354 (25.9)	434 (32.2)	536 (42.4)	657 (52.6)
Phosphorus (mg/day)	1205.8 (463.2)	1048.1 (411.3)	1339.3 (491.7)	1084.1 (413.7)	552 (40.4)	627 (46.6)	529 (41.9)	694 (55.6)
Manganese (mg/day)	3.5 (1.2)	3.2 (1.0)	3.8 (1.4)	3.3 (1.3)	515 (37.7)	603 (44.8)	535 (42.4)	534 (42.8)
Nutrients with DG								
Protein, % energy (%/day)	13.8 (3.1)	13.9 (2.8)	14.0 (3.8)	13.8 (3.1)	459 (33.6)	439 (32.6)	511 (40.5)	464 (37.1)
Fat, % energy (%/day)	29.9 (6.8)	30.5 (6.6)	29.8 (8.7)	30.1 (7.4)	817 (59.8)	854 (63.4)	694 (54.9)	740 (59.2)
SFA, % energy (%/day)	9.9 (2.6)	10.2 (2.4)	10.0 (3.2)	10.1 (2.8)	680 (49.7)	739 (54.9)	625 (49.5)	642 (51.4)
Carbohydrate, % energy (%/day)	52.2 (10.8)	50.7 (11.8)	55.6 (15.2)	52.5 (11.6)	472 (34.5)	485 (36.0)	440 (34.8)	489 (39.2)
Dietary fiber (g/day)	11.7 (4.8)	10.7 (4.4)	12.1 (5.0)	10.8 (4.4)	906 (66.3)	994 (73.8)	1096 (86.8)	1100 (88.1)
Sodium, g salt equivalent (g/day)	11.9 (3.3)	10.6 (3.1)	12.4 (3.6)	10.8 (3.1)	1323 (96.7)	1280 (95.1)	1217 (96.4)	1181 (94.6)
Potassium (mg/day)	2441.5 (984.1)	2189.1 (877.6)	2662.9 (1065.0)	2241.8 (905.3)	549 (40.2)	579 (43.0)	649 (51.4)	779 (62.4)

*Note:* Data are presented as mean (±standard deviation).

Abbreviations: AI, adequate intake; DG, dietary goal; EAR, estimated average requirement.

^a^Energy requirement based on a moderate level of physical activity.

^b^Retinol activity equivalents.

**Table 4:** Energy intake, macronutrient proportions, and dietary fiber by daily breakfast consumption intake status.

Nutrient intakes	Daily breakfast consumers *N* = 4891	Nondaily breakfast consumers *N* = 334	*p*
Energy (kcal/day)	2122.6 (597.4)	1969.4 (638.9)	**< 0.001**
Fat, % energy (%/day)	30.1 (7.1)	30.4 (10.6)	0.599
Protein, % energy (%/day)	13.9 (3.1)	13.6 (4.7)	**< 0.001**
Carbohydrate, % energy (%/day)	52.5 (12.0)	55.3 (18.6)	**0.013**
Dietary fiber (g/day)	11.4 (4.6)	10.0 (5.6)	**< 0.001**

*Note:* Data are presented as mean (±standard deviation). Significant differences are highlighted in bold (*p* < 0.05).

**Table 5:** Energy intake, macronutrient proportions, and dietary fiber by body weight status.

Nutrient intakes	Thinness *N* = 632	Normal weight *N* = 3932	Overweight *N* = 548	Obesity *N* = 113	*p*
Energy (kcal/day)	2048.7 (573.8)	2130.7 (600.0)	2062.4 (626.1)	2093.2 (637.7)	**0.001**
Fat, % energy (%/day)	29.9 (7.6)	30.1 (7.5)	30.1 (6.8)	31.5 (6.7)	0.302
Protein, % energy (%/day)	13.6 (3.0)	13.9 (3.2)	14.2 (3.2)	15.0 (4.1)	**0.002**
Carbohydrate, % energy (%/day)	51.6 (12.0)	52.9 (12.7)	52.0 (11.9)	54.4 (12.2)	0.160
Dietary fiber (g/day)	10.9 (4.6)	11.5 (4.7)	10.8 (4.5)	11.0 (4.6)	**< 0.001**

*Note:* Data are presented as mean (±standard deviation). Significant differences are highlighted in bold (*p* < 0.05).

## Data Availability

Data cannot be shared for privacy or ethical reasons.

## Supporting Information

Supporting Table 1: Reference values from the 2025 Japanese Dietary Guidelines by sex and age group (10-11 and 12–14 years).

## References

[1] (2024). *Adolescent Health*.

[2] Blum R. W., Astone N. M., Decker M. R., Mouli V. C. (2014). A Conceptual Framework for Early Adolescence: A Platform for Research. *International Journal of Adolescent Medicine and Health*.

[3] Saavedra J. M., Prentice A. M. (2023). Nutrition in School-Age Children: A Rationale for Revisiting Priorities. *Nutrition Reviews*.

[4] Klonizakis M., Bugg A., Hunt B., Theodoridis X., Bogdanos D. P., Grammatikopoulou M. G. (2021). Assessing the Physiological Effects of Traditional Regional Diets Targeting the Prevention of Cardiovascular Disease: A Systematic Review of Randomized Controlled Trials Implementing Mediterranean, New Nordic, Japanese, Atlantic, Persian and Mexican Dietary Interventions. *Nutrients*.

[5] Shinsugi C., Takimoto H. (2023). Trends in Mean Energy and Nutrient Intakes in Japanese Children and Adolescents: The National Health and Nutrition Survey, 1995–2019. *Nutrients*.

[6] Sato Y., Miyanaga M., Wang D.-H. (2020). Psychosocial Determinants of Fruit and Vegetable Intake in Japanese Adolescents: A School-Based Study in Japan. *IJERPH*.

[7] Yang Y., Yuan S., Liu Q. (2022). Meeting 24-Hour Movement and Dietary Guidelines: Prevalence, Correlates and Association With Weight Status Among Children and Adolescents: A National Cross-Sectional Study in China. *Nutrients*.

[8] Jenkins M., Jefferds M. E. D., Aburto N. J., Ramakrishnan U., Martorell R., Addo O. Y. (2023). What Do United States Adolescents Eat? Food Group Consumption Patterns and Dietary Diversity From a Decade of Nationally Representative Data. *Current Developments in Nutrition*.

[9] Horikawa C., Murayama N., Ishida H. (2020). Nutrient Adequacy of Japanese Schoolchildren on Days With and Without a School Lunch by Household Income. *Food & Nutrition Research*.

[10] Asakura K., Sasaki S. (2017). School Lunches in Japan: Their Contribution to Healthier Nutrient Intake Among Elementary-School and Junior High-School Children. *Public Health Nutrition*.

[11] Deka M.Kr., Malhotra A., Yadav R., Gupta S. (2015). Dietary Pattern and Nutritional Deficiencies Among Urban Adolescents. *Journal of Family Medicine and Primary Care*.

[12] Ikeda I., Fujihara K., Morikawa Yoshizawa S. (2024). Association Between Screen Time, Including That for Smartphones, and Overweight/Obesity Among Children in Japan: NICE EVIDENCE Study 4. *Endocrine Journal*.

[13] Cole T. J., Lobstein T. (2012). Extended International (IOTF) Body Mass Index Cut-Offs for Thinness, Overweight and Obesity. *Pediatric Obesity*.

[14] Oshima Y., Hikihara Y., Kasanami R., Murase N., Ishii K. (2017). Validity of Moderate to Vigorous Physical Activity According to the Modified Version of the International Physical Activity Questionnaire for Japanese Early Adolescents. *Japanese Journal of Physical Fitness and Sports Medicine*.

[15] Kobayashi S., Murakami K., Sasaki S. (2011). Comparison of Relative Validity of Food Group Intakes Estimated by Comprehensive and Brief-Type Self-Administered Diet History Questionnaires Against 16 D Dietary Records in Japanese Adults. *Public Health Nutrition*.

[16] Watanabe T., Kawai R. (2018). Advances in Food Composition Tables in Japan-Standard Tables of Food Composition in Japan—2015—(Seventh Revised Edition). *Food Chemistry*.

[17] Report of the Study Group on the Formulation of the Dietary Reference Intakes for Japanese (2025 Edition). https://www.mhlw.go.jp/stf/newpage_44138.html.

[18] Shinozaki N., Murakami K., Masayasu S., Sasaki S. (2023). Usual Nutrient Intake Distribution and Prevalence of Nutrient Intake Inadequacy Among Japanese Children and Adults: A Nationwide Study Based on 8-Day Dietary Records. *Nutrients*.

[19] Walsh N. M., Flynn A., Walton J., Kehoe L. (2024). Optimal Growth and Development: Are Teenagers Getting Enough Micronutrients From Their Diet?. *Proceedings of the Nutrition Society*.

[20] Yim J.-E., Choi H.-N., Park J.-S., Ahmed K. (2022). A Comparison of Dietary Quality Among Korean Obese Children and Adolescents. *Current Developments in Nutrition*.

[21] Figueiredo Moreira C. F., Cople-Rodrigues C. D. S., Giannini D. T., Kuschnir M. C. C., De Oliveira C. L. (2020). Low Intake of Dietary Fibre Among Brazilian Adolescents and Association With Nutritional Status: Cross-Sectional Analysis of Study of Cardiovascular Risks in Adolescents Data. *Public Health Nutrition*.

[22] Shinsugi C., Takimoto H. (2025). Food Consumption Trends in Japanese Children and Adolescents: The National Health and Nutrition Survey, 2001–2019. *Foods*.

[23] Martini D., Godos J., Bonaccio M., Vitaglione P., Grosso G. (2021). Ultra-Processed Foods and Nutritional Dietary Profile: A Meta-Analysis of Nationally Representative Samples. *Nutrients*.

[24] Barr S. I., DiFrancesco L., Fulgoni V. L. (2014). Breakfast Consumption Is Positively Associated With Nutrient Adequacy in Canadian Children and Adolescents. *British Journal of Nutrition*.

[25] Jones L., Ness A., Emmett P. (2021). Misreporting of Energy Intake From Food Records Completed by Adolescents: Associations With Sex, Body Image, Nutrient, and Food Group Intake. *Frontiers in Nutrition*.

[26] Zhang Y., Ojima T., Murata C. (2007). Calcium Intake Pattern Among Japanese Women Across Five Stages of Health Behavior Change. *Journal of Epidemiology*.

[27] Asakura K., Etoh N., Imamura H. (2020). Vitamin D Status in Japanese Adults: Relationship of Serum 25-Hydroxyvitamin D with Simultaneously Measured Dietary Vitamin D Intake and Ultraviolet Ray Exposure. *Nutrients*.

[28] Wu S.-E., Chen W.-L. (2022). Moderate Sun Exposure Is the Complementor in Insufficient Vitamin D Consumers. *Frontiers in Nutrition*.

[29] Zhang S., Otsuka R., Tomata Y. (2019). A Cross-Sectional Study of the Associations Between the Traditional Japanese Diet and Nutrient Intakes: The NILS-LSA Project. *Nutrition Journal*.

[30] Tomata Y., Zhang S., Kaiho Y., Tanji F., Sugawara Y., Tsuji I. (2019). Nutritional Characteristics of the Japanese Diet: A Cross-Sectional Study of the Correlation Between Japanese Diet Index and Nutrient Intake Among Community-Based Elderly Japanese. *Nutrition*.

[31] Perez V., Chang E. T. (2014). Sodium-to-Potassium Ratio and Blood Pressure, Hypertension, and Related Factors. *Advances in Nutrition*.

[32] Fujiwara S., Harada K., Hagiya H. (2024). Trends in Childhood Obesity in Japan: A Nationwide Observational Study From 2012 to 2021. *Clinical Obesity*.

[33] EFSA Panel on Dietetic Products Nutrition and Allergies NDA (2010). Scientific Opinion on Dietary Reference Values for Carbohydrates and Dietary Fibre. *EFS2*.

[34] Katagiri R., Goto A., Sawada N. (2020). Dietary Fiber Intake and Total and Cause-Specific Mortality: The Japan Public Health Center-Based Prospective Study. *The American Journal of Clinical Nutrition*.

[35] Cribb V., Emmett P., Northstone K. (2013). Dietary Patterns Throughout Childhood and Associations With Nutrient Intakes. *Public Health Nutrition*.

[36] Winzer E., Wakolbinger M., Schätzer M. (2021). Impact of a Nutrition Education Programme on Free Sugar Intake & Nutrition-Related Knowledge in Fifth-Grade Schoolchildren. *The European Journal of Public Health*.

[37] Jeans M. R., Landry M. J., Vandyousefi S. (2023). Effects of a School-Based Gardening, Cooking, and Nutrition Cluster Randomized Controlled Trial on Unprocessed and Ultra-Processed Food Consumption. *The Journal of Nutrition*.

