# Supplementary material for: Nutrient Inadequacy Rates Among Japanese Adolescents Aged 10–14: Cross-Sectional Pooled Analysis From 2018 to 2023 (NICE EVIDENCE Study 5)
Source: J Nutr Metab. 2025 Jul 24;2025:5568303. doi: 10.1155/jnme/5568303 (PMC12313374; doi:10.1155/jnme/5568303)
Supplement: Supporting Information — Additional supporting information can be found online in the Supporting Information section. [file 5568303.f1.docx]

Supplementary tables

| Supplementary Table 1. Reference values from the 2025 Japanese Dietary Guidelines by sex and age group (10–11 and 12–14 years) | | | | |
| --- | --- | --- | --- | --- |
| Nutrient | Male 10–11y Intake | Female 10–11y | Male 12–14y | Female 12–14y |
| **Energy**^a^ (kcal/day) | 2250 | 2100 | 2600 | 2400 |
| **Nutrients without DRI** |  |  |  |  |
| Fat (g/day) | - | - | - | - |
| SFA (g/day) | - | - | - | - |
| Carbohydrate (g/day) | - | - | - | - |
| Sodium (mg/day) | - | - | - | - |
| **Nutrients with EAR** |  |  |  |  |
| Protein (g/day) | 40 | 40 | 50 | 45 |
| Vitamin A^b^ (µg/day) | 450 | 400 | 550 | 500 |
| Vitamin B_1_ (mg/day) | 0.7 | 0.6 | 0.8 | 0.7 |
| Vitamin B_2_ (mg/day) | 1.1 | 1.1 | 1.3 | 1.2 |
| Niacin (mgNE^c^/day) | 11 | 10 | 12 | 12 |
| Vitamin B_6_ (mg/day) | 0.9 | 1.0 | 1.2 | 1.1 |
| Vitamin B_12_ (µg/day) | 3.0 | 3.0 | 4.0 | 4.0 |
| Folate (µg/day) | 150 | 150 | 190 | 190 |
| Vitamin C (mg/day) | 60 | 60 | 75 | 75 |
| Calcium (mg/day) | 600 | 600 | 850 | 700 |
| Magnesium (mg/day) | 180 | 180 | 250 | 240 |
| Iron (mg/day) | 6.5 | 6.5 (Non-menstrual criteria) | 7.5 | 6.5 (Non-menstrual criteria) |
|  |  | 8.5 (Menstrual criteria) |  | 9.0 (Menstrual criteria) |
| Zinc (mg/day) | 5.5 | 5.5 | 7.0 | 6.5 |
| Copper (mg/day) | 0.5 | 0.5 | 0.7 | 0.6 |
| **Nutrients with AI** |  |  |  |  |
| n-6 PUFA (g/day) | 9.0 | 9.0 | 11.0 | 11.0 |
| n-3 PUFA (g/day) | 1.7 | 1.7 | 2.2 | 1.7 |
| Vitamin D (µg/day) | 8.0 | 8.0 | 9.0 | 9.0 |
| Vitamin E (mg/day) | 5.0 | 5.5 | 6.5 | 6.0 |
| Vitamin K (µg/day) | 110 | 130 | 140 | 150 |
| Pantothenic acid (mg/day) | 6.0 | 6.0 | 7.0 | 6.0 |
| Potassium (mg/day) | 1900 | 1800 | 2400 | 2200 |
| Phosphorus (mg/day) | 1100 | 1000 | 1200 | 1100 |
| Manganese (mg/day) | 3.0 | 3.0 | 3.5 | 3.0 |
| **Nutrients with DG** |  |  |  |  |
| Protein, % energy | 13–20 | 13–20 | 13–20 | 13–20 |
| Fat, % energy | 20–30 | 20–30 | 20–30 | 20–30 |
| SFA, % energy | 10 or below | 10 or below | 10 or below | 10 or below |
| Carbohydrate, % energy | 50–65 | 50–65 | 50–65 | 50–65 |
| Dietary fiber (g/day) | 13 or above | 13 or above | 17 or above | 16 or above |
| Sodium, g salt equivalent (g/day) | 6.0 | 6.0 | 7.0 | 6.5 |
| Potassium (mg/day) | 2200 | 2000 | 2600 | 2400 |
| EAR: Estimated Average Requirement; AI: Adequate Intake; DG: Dietary Goal. ^a^Energy requirement based on moderate level of physical activity; ^b^Retinol activity equivalents; ^c^ Niacin equivalent. | | | | |
